# Blood Pressure Control and Cardiovascular Outcomes: Real-world Implications of the 2017 ACC/AHA Hypertension Guideline

**DOI:** 10.1038/s41598-018-31549-5

**Published:** 2018-09-03

**Authors:** Ji Hyun Lee, Sun-Hwa Kim, Si-Hyuck Kang, Jun Hwan Cho, Youngjin Cho, Il-Young Oh, Chang-Hwan Yoon, Hae-Young Lee, Tae-Jin Youn, In-Ho Chae, Cheol-Ho Kim

**Affiliations:** 1 Department of Cardiology, Wonju Medical College, Yonsei University, Wonju, Korea; 2 Cardiovascular Center, Seoul National University Bundang Hospital, Seongnam-si, Korea; 3 Department of Internal Medicine, Seoul National University, Seoul, Korea; 4 Cardiovascular Center, Seoul National University Hospital, Seoul, Korea

## Abstract

The 2017 American College of Cardiology/American Heart Association (ACC/AHA) hypertension guideline lowered the threshold defining hypertension and treatment target from 140/90 mmHg to 130/80 mmHg. We compared the 2017 ACC/AHA guideline and the Eighth Joint National Committee (JNC8) report with regard to the current status of hypertension using the Korean National Health and Nutrition Examination Survey. The association between blood pressure (BP) control and long-term major cardiovascular outcomes (MACEs) was analyzed using the Korea National Health Insurance Service cohort. In the cross-sectional study with 15,784 adults, the prevalence of hypertension was expected to be 49.2 ± 0.6% based on the definition suggested by the 2017 ACC/AHA guideline versus 30.4 ± 0.6% based on the JNC8 report. In a longitudinal analysis with 373,800 hypertensive adults for the median follow-up periods of 11.0 years, the adults meeting the target goal BP goal of 2017 ACC/AHA guideline were associated with 21% reduced risk of MACEs compared with adults, not meeting 2017 ACC/AHA BP goal but meeting JNC8 target goal. In conclusion, substantial increase of prevalence of hypertension is expected by the 2017 ACC/AHA guideline. This study also suggests endorsing the aggressive approach would lead to an improvement in cardiovascular care.

## Introduction

Elevated blood pressure (BP) is the leading cause of mortality and disease burden^1,2^. The World Health Organization estimates that 40% of adults worldwide have hypertension^3^. Elevated systolic BP was responsible for 10.5 million deaths globally in 2016, and the burden from high BP is continuously increasing^2^. Elevated BP has been shown to account for 58.3% of deaths from hemorrhagic stroke and 54.5% of deaths from ischemic heart disease^4^.

Epidemiologic studies have suggested that the relationship between BP and cardiovascular risks exists as low as 110–115 mmHg for systolic BP and 70–75 mmHg for diastolic BP^4^. Every 20 mmHg systolic or 10 mmHg diastolic BP increase above the threshold has shown to double the risk of mortality from ischemic heart disease and stroke^5^. However, for decades, guidelines generally defined hypertension as a BP ≥ 140/90 mmHg, and suggested BP should be maintained at <140/90 mmHg^6–9^. The newly released 2017 American College of Cardiology/American Heart Association (ACC/AHA) Guideline for the Prevention, Detection, Evaluation, and Management of High Blood Pressure in Adults supports a more aggressive diagnostic and treatment approach^10^. The new guideline defines hypertension as a BP ≥ 130/80 mmHg and recommends hypertensive patients to maintain their BP under <130/80 mmHg.

The impact of the new guideline is expected to be immense. Muntner *et al*. used the data from a nationwide survey in the United States to show that the prevalence of hypertension increased from 31.9% to 45.6% when applying the 2017 ACC/AHA guideline^11^. The radical changes in the recommendations have sparked fierce debates among researchers and practitioners^12–14^. In this study, we sought to estimate the potential impact of the new hypertension guideline on clinical practice in Korea. We specifically attempted to determine the following: how many people would be classified as having hypertension, how many among them would be recommended pharmacologic treatment, and whether more aggressive BP goals would lead to better cardiovascular outcomes.

## Methods

### Data sources and design: Korea National Health and Nutrition Examination Survey (KNHANES)

Data from KNHANES were used to estimate the current status of hypertension prevalence and control. KNHANES is a national surveillance constructed to assess the health and nutritional status in Korean population^15^. Detailed information on socioeconomic status, health and dietary behaviors, quality of life, healthcare utilization, anthropometric measures, and biochemical profiles are included in the database. Subjects representative of the Korean population were chosen for this study using a complex, stratified, multistage, cluster sampling method. BPs were measured three times on the subjects’ right arms using an appropriately sized arm cuff and mercury sphygmomanometer (Baumanometer; WA Baum Co., New York, NY, USA) after the subject rested in a seated position for at least 5 minutes. We calculated the final BP value from the average of the second and third measurements^16^. The sample weights were constructed by accounting for the complex survey design, survey non-response, and post-stratification. Among a total of 22,948 subjects who participated in the KNHANES VI between 2013 and 2015 (8,018 in 2013, 7,550 in 2014, and 7,380 in 2015), 15,784 adults aged 30 years or older were analyzed in this study.

### Data sources and design: National Health Insurance System–National Health Screening Cohort (NHIS-HEALS)

We selected hypertensive subjects from the NHIS-HEALS cohort^17^. The National Health Insurance Service is the single insurance provider in Korea covering all citizens. Enrollees in the insurance system are entitled to standardized medical examinations biennially, including standardized self-reporting questionnaires on lifestyle, medical history, height, weight, blood pressure measurements, and laboratory tests. The cohort contains data of 514,866 subjects who underwent routine check-ups between 2002 and 2003, and includes demographic data, eligibility status, income levels, medical claims, and death records through the end of 2013. The Seoul National University Hospital Institutional Review Board approved this study (I-2018-150) and waived the mandate for obtaining informed consents. This study was conducted according to the relevant guidelines and national regulations.

### Definitions

The prevalence of hypertension and the percentage of adults requiring antihypertensive drug treatment were compared with 2017 ACC/AHA guideline and Eighth Joint National Committee (JNC8) report^8^. The 2017 ACC/AHA guideline defines hypertension as a systolic BP ≥ 130 mmHg or a diastolic BP ≥ 80 mmHg, while previous guidelines, including the JNC8, have defined hypertension as a BP of 140/90 mmHg or higher^6–9^. The thresholds for pharmacologic antihypertensive treatment based on the guidelines are summarized in Supplementary Table S1. The subjects who were already taking antihypertensive medications were also regarded as having hypertension and meeting both criteria for medical treatment. The 2017 ACC/AHA guideline recommends a target BP < 130/80 mmHg, while the previous guideline recommended a BP < 140/90 mmHg except in the elderly (systolic BP < 150 mmHg).

The data of patients with hypertension and follow-up BP records were extracted from the NHIS-HEALS cohort. Subjects were considered as having hypertension if: (a) hypertension was diagnosed once or more during a hospitalization, or at two or more outpatient clinic visits, (b) antihypertensive medications were prescribed for >6 months, or (c) BP readings were 130/80 mmHg or above^18^. Any subjects who had experienced myocardial infarction (MI), heart failure (HF), or stroke before the diagnosis of hypertension were excluded from this analysis. Then, the study subjects were categorized into 3 groups according to BP levels achieved: (a) achieved BP below the target BP goal recommended by the 2017 ACC/AHA guidelines (2017 ACC/AHA group), (b) achieved BP below the target BP goal recommended by the JNC8 guidelines but above that of the 2017 ACC/AHA guidelines (JNC8 group), and (c) achieved BP above the goals provided by both guidelines (poor group).

Study subjects were followed up until the occurrence of the primary endpoint, until death, or until the end of the cohort (December 31, 2013). Vital status was confirmed using the national administrative death records. The primary endpoint was major cardiovascular events, comprising a composite of cardiovascular death, MI, HF, and stroke. The secondary endpoints included all cause death and each separate component of the primary endpoint.

### Statistical analysis

Data were presented as mean ± standard error or % (number). Weights based on the complex sampling design of KNHANES were used for all the statistical analyses to avoid biased estimates^15^. The variables representing strata, cluster, and weight were included in the raw data. Cox proportional hazard models were used to evaluate the associations between levels of BP control and the occurrence of major cardiovascular events. We used restricted cubic spline curves to visualize the relationship between the independent and dependent variables using the ‘rms’ packages in the R program. We performed multivariable regression analyses with adjustments for age, sex, baseline systolic BP, baseline diastolic BP, body mass index, diabetes, dyslipidemia, malignancy, renal disease, liver disease, chronic pulmonary disease, rheumatic disease, and smoking status. Propensity score matching was performed using the aforementioned variables. A nearest match algorithm was constructed in 1:1 matching ratios using ‘MatchIt’ packages in the R program. Statistical analyses were conducted using SPSS Statistics (IBM Corp., Armonk, NY, USA) and R programming version 3.2.4 (http://www.R-project.org; The R Foundation for Statistical Computing, Vienna, Austria).

## Results

### Potential impact of the new hypertension definition

**Figure 1:**
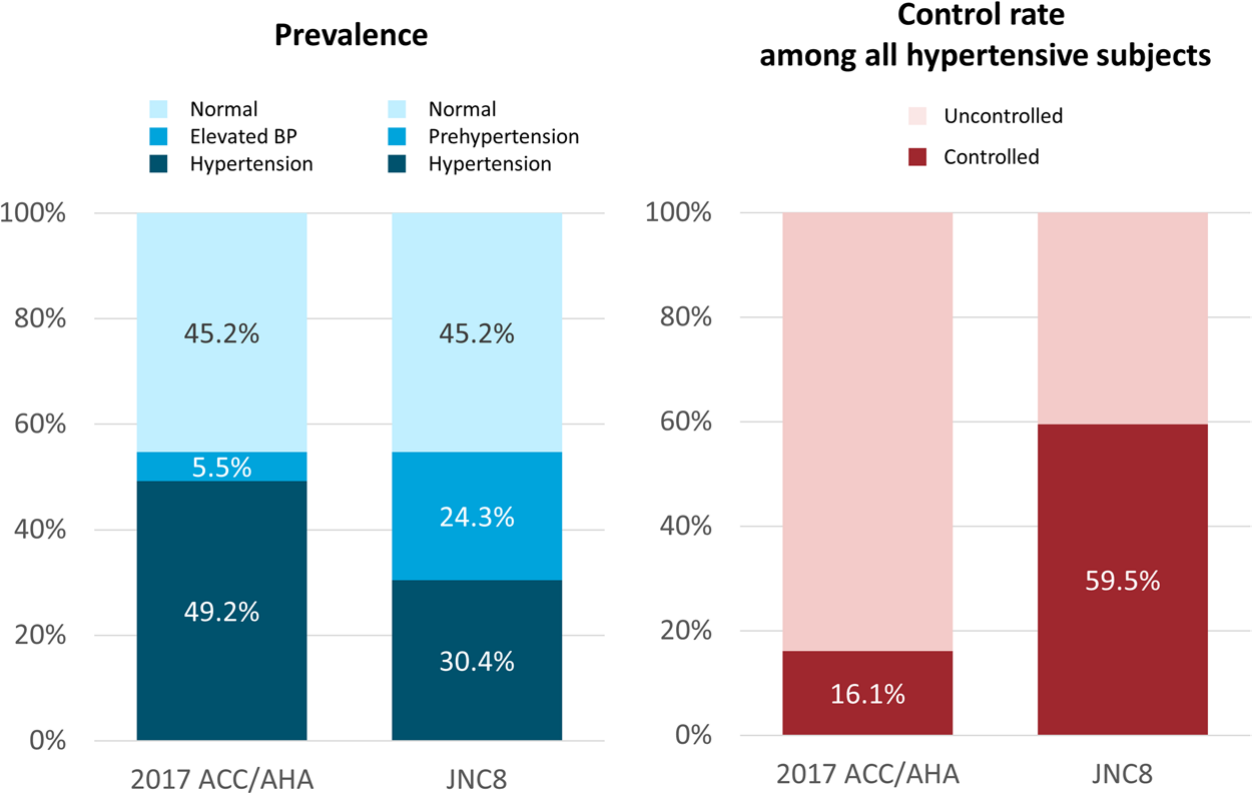
The potential impact of implementing the 2017 ACC/AHA guideline for high blood pressure (BP) based on hypertension prevalence (left panel) and hypertension control (right panel). The 2017 ACC/AHA: 2017 American College of Cardiology/American Heart Association Guideline for the Prevention, Detection, Evaluation and Management of High Blood Pressure in Adults; JNC8: Evidence-Based Guideline for the Management of High Blood Pressure in Adults: Report from the Panel Members Appointed to the Eighth Joint National Committee.

The prevalence of hypertension was estimated to be 49.2% based on the BP criteria suggested by the 2017 ACC/AHA guidelines, which was a significant increase compared to the 30.4% prevalence rate based on the previous definitions (Fig. 1). A total of 5.6 million Koreans who were considered to have pre-hypertension were classified as hypertensive based on the new criteria. The increase was more prominent among men and younger age groups, whereas it was consistent across income levels, areas of residence, and education levels (see Supplementary Table S2).

The profiles of the subjects in each hypertension category were described in Supplementary Table S3. The risk profiles of the 5.6 million subjects (18.8% of adults) who were newly classified as having stage 1 hypertension based on the 2017 ACC/AHA guidelines (systolic BP 130–139 mmHg or diastolic BP 80–89 mmHg) were between those of stage 2 hypertension and normal BP groups. The estimated 10-year atherosclerotic cardiovascular disease risk was 8.2%.

As the 2017 ACC/AHA guideline suggested lower BP threshold for hypertension to underline the importance of the early initiation of non-pharmacological treatment, medical treatment was indicated partly in the subjects with newly classified hypertension. BP-lowering medications are recommended in 35.3% of adults by the 2017 ACC/AHA guidelines. This is in contrast with the JNC8 guideline which showed a small difference between the prevalence of hypertension (30.4%) and the proportion of subjects requiring medical treatment (29.4%). A total of 1.8 million subjects (6.0% of Korean adults) were recommended to newly start antihypertensive medications based on the new recommendations (see Supplementary Table S2). Among various indications, the presence of cardiovascular disease and high risk of atherosclerotic cardiovascular disease were the predominant indications (see Supplementary Fig. S1).

### Potential impact of the new BP control target

The hypertension control rate used to be 59.5% in Korea when using the target BP of JNC8 guideline. However, if the 2017 ACC/AHA guidelines were applied, percentage of adults meeting the target BP goal would have decreased to 16.1% (see Supplementary Table S4). This was due to both an increase in the number of hypertensive subjects and the more stringent target BP goal.

### Level of BP control and risk of cardiovascular events

We extracted follow-up data spanning a median of 11.0 years from the NHIS-HEALS Cohort. The data were obtained from a total of 373,800 hypertensive individuals with no previous history of major cardiovascular events. The baseline risk profiles represented the primary prevention population: 58% men, mean age of 56.8 years, baseline BP of 140/88 mmHg, body mass index of 24.3 kg/m^2^, and a low prevalence of comorbidities (Table 1).

**Table 1:** Comparison of baseline risk factors of study subjects stratified by blood pressure (BP) control levels.

	All adults with hypertension	Below 2017 ACC/AHA target	Below JNC8 but above 2017 ACC/AHA target	Above JNC8 target	P value
Number of subjects (%)	373,800 (100.0%)	96,317 (25.8%)	145, 981 (39.0%)	131, 502 (35.2%)	
Age, years	56.8 ± 9.5 (373,800)	56.6 ± 9.2 (96,317)	57.1 ± 9.6 (145,981)	56.7 ± 9.5 (131,502)	0.275
Female sex, %	42.0% (157,005/373,800)	46.7% (44,992/96,317)	41.6% (60,700/145,981)	39% (51,313/131,502)	<0.001
Body mass index, kg/m^2^	24.3 ± 3.0 (373,664)	23.7 ± 2.9 (96,283)	24.3 ± 2.9 (145,945)	24.8 ± 3.0 (131,436)	<0.001
**Blood pressure**					
Baseline SBP, mmHg	140.4 ± 12.4 (340,776)	136.2 ± 9.1 (74,832)	137.5 ± 9.9 (135,669)	145.9 ± 14.4 (130,275)	<0.001
Baseline DBP, mmHg	87.8 ± 8.1 (340,776)	85.3 ± 6.4 (74,832)	85.9 ± 6.8 (135,669)	91.2 ± 8.9 (130,275)	<0.001
Achieved SBP, mmHg	131.4 ± 17.2 (373,800)	113.6 ± 8.7 (96,317)	128.4 ± 8.0 (145,981)	147.7 ± 14.4 (131,502)	<0.001
Achieved DBP, mmHg	81.7 ± 11.0 (373,800)	69.5 ± 5.6 (96,317)	80.3 ± 4.5 (145,981)	92.3 ± 8.8 (131,502)	<0.001
**Comorbidities, %**					
Diabetes	6.6% (24,733/373,800)	6.9% (6,631/96,317)	5.7% (8,307/145,981)	7.4% (9,795/131,502)	<0.001
Dyslipidemia	17.5% (65,541/373,800)	22.0% (21,183/96,317)	17.7% (25,811/145,981)	14.1% (18,547/131,502)	<0.001
Current smoking	21.2% (76,062/358,407)	19.8% (18,392/92,714)	20.9% (29,257/140,043)	22.6% (28,413/125,650)	<0.001
Malignancy	4.4% (16,432/373,800)	6.2% (5,987/96,317)	4.6% (6,659/145,981)	2.9% (3,786/131,502)	<0.001
Renal disease	0.5% (2,029/373,800)	0.7% (666/96,317)	0.5% (696/145,981)	0.5% (667/131,502)	<0.001
Liver disease	0.5% (1,731/373,800)	0.7% (694/96,317)	0.5% (668/145,981)	0.3% (369/131,502)	<0.001
Chronic pulmonary disease	26.2% (97,919/373,800)	33.0% (31,787/96,317)	27.5% (40,142/145,981)	19.8% (25,990/131,502)	<0.001
Rheumatic disease	6.5% (24,483/373,800)	8.7% (8,382/96,317)	6.7% (9,833/145,981)	4.8% (6,268/131,502)	<0.001
**Laboratory findings**					
Total cholesterol, mg/dL	201.5 ± 38.1 (373,323)	197.3 ± 37.1 (96,180)	201.3 ± 37.6 (145,816)	204.9 ± 39.0 (131,327)	<0.001
HDL cholesterol, mg/dL	54.4 ± 28.3 (332,700)	54.8 ± 27.5 (88,600)	54.4 ± 28.2 (131,619)	54.0 ± 29.0 (112,481)	0.037
LDL cholesterol, mg/dL	117.4 ± 38 (330,722)	118.4 ± 37.2 (88,224)	118.2 ± 37.8 (130,835)	115.7 ± 38.9 (111,663)	<0.001
Triglyceride, mg/dL	145.1 ± 93.6 (332,428)	134.5 ± 86.4 (88,548)	144.3 ± 91.8 (131,517)	154.5 ± 99.9 (112,363)	<0.001
Serum creatinine, mg/dL	1.1 ± 1.3 (332,713)	1.0 ± 1.1 (88,602)	1.1 ± 1.3 (131,623)	1.1 ± 1.4 (112,488)	0.819

Values were presented as mean ± SD or percent with numbers of subjects. 2017 ACC/AHA: 2017 American College of Cardiology/American Heart Association Guideline for the Prevention, Detection, Evaluation and Management of High Blood Pressure in Adults; DBP: diastolic BP; HDL: high density lipoprotein; JNC8: Evidence-Based Guideline for the Management of High Blood Pressure in Adults: Report from the Panel Members Appointed to the Eighth Joint National Committee; LDL: low density lipoprotein; SBP: systolic BP.

**Figure 2:**
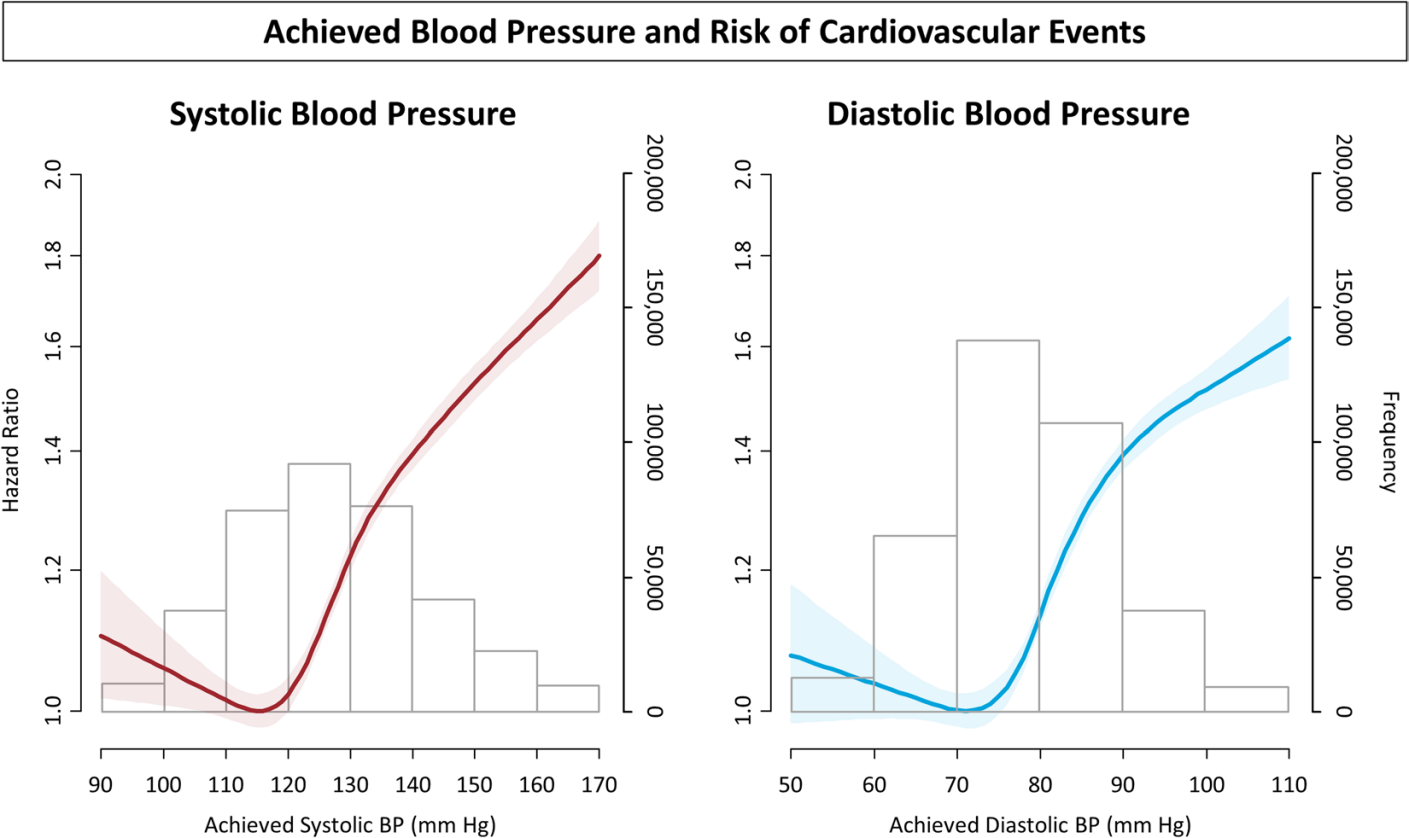
Restricted cubic spline curve of achieved blood pressure and adjusted risk of cardiovascular events: (left) systolic blood pressure, and (right) diastolic blood pressure. Curves represent adjusted hazard ratios (solid line) and the 95% confidence intervals (shades). The distributions of achieved systolic and diastolic blood pressure are also shown (bars).

The overall annualized 10-year cardiovascular event rate was 7.9%. Figure 2 shows the nonlinear relationship between follow up BP levels and the risk of cardiovascular events. The nadir was at 115 mmHg for systolic BPs and 70 mmHg for diastolic BPs. Every 20 and 10 mmHg increase (above 115 and 70 mmHg) in systolic and diastolic BP was associated with an increased risk by 29.2% and 19.3%, respectively. The slope was most steep at 120‒140 mmHg for systolic BP and at 75‒90 mmHg for diastolic BP. Kaplan-Meier survival curves stratified by follow up BP values are shown in Supplementary Fig. S2.

### 2017 ACC/AHA guideline vs. JNC8 guideline

**Figure 3:**
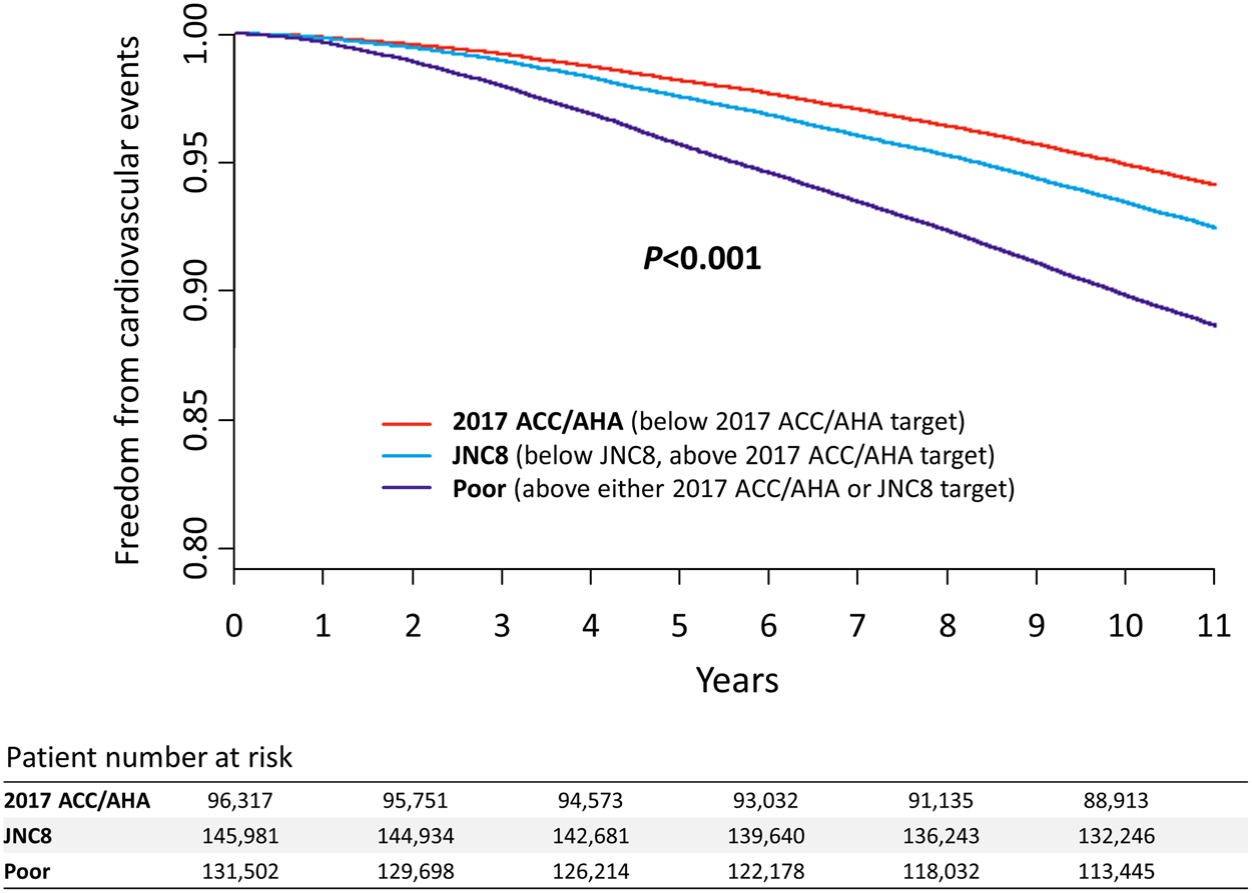
Kaplan-Meier survival curve free from major cardiovascular events among hypertensive subjects according to their achieved blood pressure. The red line indicates subjects who achieved the target blood pressure recommended by the 2017 ACC/AHA guidelines (2017 ACC/AHA group); the blue line indicates subjects who achieved the target blood pressure recommended by the JNC8 guidelines but not the 2017 ACC/AHA guidelines (JNC8 group); the purple line indicates subjects who achieved neither target goal (poor group).

The baseline characteristics of the 3 study groups classified based on the follow up BP levels (the 2017 ACC/AHA, JNC8, and poor groups) are compared in Table 1. Whereas age did not differ significantly between the groups, there was a trend toward a higher prevalence in men and higher risk profiles among the less stringently defined groups. Survival free from the primary endpoint, a composite cardiovascular death, MI, HF, and stroke, is depicted in Fig. 3. The 10-year annualized rates of the primary endpoint were 5.5%, 7.1%, and 10.8% for the 2017 ACC/AHA, JNC8, and poor groups, respectively (Table 2). The 2017 ACC/AHA group showed a 23% reduced risk compared to the JNC8 group (hazard ratio [HR], 0.77; 95% confidence intervals [CI], 0.75‒0.80), and a 50% reduced risk compared to the poor group (HR, 0.50; 95% CI, 0.49‒0.52). This finding was consistent across each component of the primary endpoint, but was greater for stroke. Models adjusted for multivariable and matched with propensity score (see Supplementary Fig. S3) also confirmed these observations. A subgroup analysis revealed that the benefits of stricter BP control were consistent regardless of the presence of diabetes or chronic kidney disease (see Supplementary Fig. S4).

**Table 2:** Risk of major cardiovascular events stratified by blood pressure control levels.

	10-year event rates (%)			Unadjusted analysis		Multivariable adjustment		Propensity score matching analysis	
	Below 2017 ACC/AHA target	Below JNC8 but above 2017 ACC/AHA target	Above JNC 8 target	HR (95% CI)	p-value	HR (95% CI)	p-value	HR (95% CI)	p-value
**2017 ACC/AHA goal vs. JNC8 goal**									
Composite cardiovascular events	5.47	7.06	—	0.77 (0.75‒0.80)	<0.001	0.79 (0.76‒0.82)	<0.001	0.91 (0.86‒0.97)	0.002
Death from any cause	4.66	5.48	—	0.85 (0.82‒0.88)	<0.001	0.78 (0.74‒0.81)	<0.001	0.82 (0.76‒0.88)	<0.001
Cardiac death	0.88	1.15	—	0.77 (0.71‒0.83)	<0.001	0.71 (0.64‒0.78)	<0.001	0.77 (0.66‒0.89)	<0.001
Myocardial infarction	1.16	1.44	—	0.80 (0.75‒0.86)	<0.001	0.83 (0.77‒0.91)	<0.001	0.94 (0.83‒1.06)	0.297
Stroke	2.63	3.68	—	0.72 (0.68‒0.75)	<0.001	0.74 (0.71‒0.79)	<0.001	0.89 (0.82‒0.96)	0.004
Heart failure	2.12	2.53	—	0.84 (0.79‒0.88)	<0.001	0.82 (0.77‒0.88)	<0.001	0.94 (0.86‒1.03)	0.173
**2017 ACC/AHA goal vs. poorly controlled**									
Composite cardiovascular events	5.47	—	10.84	0.50 (0.49‒0.52)	<0.001	0.55 (0.53‒0.58)	<0.001	0.52 (0.50‒0.54)	<0.001
Death from any cause	4.66	—	7.69	0.60 (0.58‒0.62)	<0.001	0.56 (0.53‒0.58)	<0.001	0.55 (0.52‒0.57)	<0.001
Cardiac death	0.88	—	1.94	0.45 (0.42‒0.49)	<0.001	0.44 (0.40‒0.49)	<0.001	0.43 (0.38‒0.48)	<0.001
Myocardial infarction	1.16	—	2.16	0.54 (0.50‒0.57)	<0.001	0.61 (0.56‒0.66)	<0.001	0.57 (0.52‒0.62)	<0.001
Stroke	2.63	—	5.90	0.44 (0.43‒0.46)	<0.001	0.53 (0.50‒0.56)	<0.001	0.49 (0.46‒0.52)	<0.001
Heart failure	2.12	—	3.66	0.58 (0.55‒0.61)	<0.001	0.59 (0.55‒0.63)	<0.001	0.56 (0.52‒0.60)	<0.001

2017 ACC/AHA: 2017 American College of Cardiology/American Heart Association Guideline for the Prevention, Detection, Evaluation and Management of High Blood Pressure in Adults; JNC8: Evidence-Based Guideline for the Management of High Blood Pressure in Adults: Report from the Panel Members Appointed to the Eighth Joint National Committee; HR: hazard ratio; CI: confidence intervals.

A sensitivity analysis was done for 231,698 hypertensive patients who were taking BP-lowering medicines. The baseline characteristics showed relatively higher risk profiles than in the main analysis population (see Supplementary Table S5). Survival analyses corroborated the study findings (see Supplementary Fig. S5 and Supplementary Table S6).

## Discussion

This study showed a substantial increase in the prevalence of hypertension (from 30.4% to 49.2%) with the lowered BP threshold of ≥130/80 mmHg suggested by the 2017 ACC/AHA guideline. However, the number of adults for whom BP-lowering medical treatment was recommended was modestly increased (from 29.4% to 35.3%). We also found that hypertensive adults meeting the target BP goal of the 2017 ACC/AHA guideline were associated with a 23% reduced risk for of major cardiovascular events compared to adults meeting the JNC8 target BP only.

The 2017 ACC/AHA guideline, which has adopted lower BP thresholds for the diagnosis of hypertension, underlines the importance of early preventive measures and non-pharmacologic interventions. Growing evidence from clinical trials and epidemiological studies suggests that lowering the BP below 140/90 mmHg may be associated with a reduced risk of adverse cardiovascular events^4,19–22^. This study showed that nearly a half of Korean adults would be diagnosed with hypertension based on the new guideline. but not all hypertensive adults would be recommended for pharmacologic treatment. A substantial proportion of them would be recommended for non-pharmacologic treatments initially. Numerically speaking, among the 5.6 million Korean adults who were newly diagnosed with hypertension, medical treatment was indicated in 1.8 million (32.2%) while non-pharmacological therapy was indicated in the remainder. Strong evidence exists supporting the efficacy and safety of non-pharmacological interventions such as weight reduction, healthy diet, sodium restriction, physical activity, and moderation in alcohol consumption^23–25^.

It has long been debated whether tighter BP control results in better clinical outcomes. The SPRINT (the Systolic Blood Pressure Intervention Trial) showed that there were significant benefits to reducing systolic BPs < 120 mmHg compared to <140 mmHg, and strongly influenced the changes introduced in the 2017 AHA/ACC guideline recommendations^20^. The trial enrolled high-risk patients (mean Framingham 10-yr cardiovascular disease risk: 24.8%); thus, it is still uncertain whether the study findings can be generalized to populations that were not included in the trial. For example, the Heart Outcomes Prevention Evaluation (HOPE)-3, which enrolled intermediate-risk hypertensive patients without previous cardiovascular disease, failed to demonstrate a significant risk reduction with intensive antihypertensive therapy^26^. In the Action to Control Cardiovascular Risk in Diabetes (ACCORD) trial, which exclusively enrolled diabetic patients and wherein the study protocol was similar with that of the SPRINT, the benefits of intensive BP control were not statistically significant^19^.

The findings of our study correspond to the current evidence supporting intensive BP control in the general population. The study subjects exhibited a low-risk profile with no previous history of major cardiovascular events, and the actual 10-year event rate was as low as 7.9%. The magnitude of the risk reduction shown in this study (23%) was comparable to that of the SPRINT (27%) and the ACCORD trial (12%). The achieved BP in this study (113.6/69.5 and 128.4/80.3 mmHg in the intensive and standard groups, respectively) was similar to, or lower than those in the SPRINT (121.4/68.7 and 134.6/76.3 mmHg) and the ACCORD trial (119.3/64.4 and 133.5/70.5 mmHg). In addition, we found no significant interactions according to the presence of diabetes or chronic renal disease.

The 2017 ACC/AHA guideline has raised practical issues. Previous studies have consistently shown poor status of hypertension control in the real world. Globally, less than a half of the subjects with hypertension are aware of the diagnosis, and less than a third of those being treated maintain BPs lower than 140/90 mmHg^27^. The control rate among all hypertensive patients in Korea is no higher than 42.9%^9^. Trying to maintain even lower BPs at <130/80 mmHg must be challenging^11^. This study also expected that tighter BP control would be required for more than 5 out of 6 Korean hypertensive patients if the new treatment targets are endorsed.

Recently, the new European Guidelines for the treatment of high BP was presented at the 2018 European Society of Hypertension meeting (The full text will be published during the upcoming European Society of Cardiology congress)^28^. The Korean Society of Hypertension also released its preliminary guideline in 2018 (written in Korean language)^29^. Both guidelines maintained the previous definition of the cut-off for hypertension of BP ≥ 140/90 mmHg. The European guideline classified systolic BP 130‒139 mmHg and/or diastolic BP 85‒89 mmHg as “high normal” in, while Korean one defined “prehypertension” as systolic BP of 130‒139 mmHg or diastolic of BP 80‒89 mmHg. However, the new guidelines commonly recommend stricter BP control than recommended in their previous versions^28,29^. In line with the 2017 ACC/AHA guideline, the target systolic BP was lowered to 130 mmHg for high risk patients, although there are variations in details across hypertensive subgroups.

This study has several limitations. First, as the National Health Insurance Service Cohort is a real-world database, BP measurements could not be standardized. Second, BPs were measured during a single visit both in the KNHANES and the cohort. Repeated visits or ambulatory measurements have been shown to minimize the chance of misclassifications^30^. The 2017 ACC/AHA guideline also stresses the role of out-of-office and self-monitored BP measurements^10^. Third, achieved BPs were defined by the mean of randomly collected BP measurements rather than repeated measurements with predefined protocols. Fourth, lifestyle intervention was not considered in this study. Lifestyle changes such as a healthy diet, sodium restriction, potassium supplementation, increased physical activity, and a reduction in alcohol consumption have been shown to be effective in lowering BP. They have also been shown to have additional health benefits beside the BP-lowering effects. Finally, there may be other confounders that were not adjust for in this study such as initiation of antihypertensive medication and statin use.

In conclusion, we estimated the potential implications and relevance of the 2017 ACC/AHA hypertension guideline in real-world clinical practices in the present study. A substantial number of adults who had not been considered hypertensive were classified as having hypertension. Approximately one third of them met the indication for pharmacologic antihypertensive treatment. Hypertensive adults meeting the target BP of 2017 ACC/AHA guideline was associated with a reduction in long-term cardiovascular events compared to those meeting the JNC8 guideline only. It can be assumed that endorsement of more aggressive BP control strategies as suggested by the 2017 ACC/AHA guideline might lead to improved cardiovascular outcomes.

## Electronic supplementary material

Supplementary information

## Data Availability

The datasets that were generated and/or analyzed during the current study are available from the corresponding author on reasonable request.
