# Supplementary material for: Blood Pressure Control and Cardiovascular Outcomes: Real-world Implications of the 2017 ACC/AHA Hypertension Guideline
Source: Sci Rep. 2018 Sep 3;8:13155. doi: 10.1038/s41598-018-31549-5 (PMC6120944; doi:10.1038/s41598-018-31549-5)

**Supplementary Information**

**Blood Pressure Control and Cardiovascular Outcomes
: Real-world Implications of the 2017 ACC/AHA Hypertension Guideline**

Ji Hyun Lee, MD, PhD^a,b^, Sun Hwa Kim, PhD^b,c^, *Si-Hyuck Kang, MD^b,c^, Jun Hwan Cho, MD^b,c^, Youngjin Cho, MD^b,c^,

Il-Young Oh, MD, PhD^b,c^, Chang-Hwan Yoon, MD, PhD^b,c^, Hae-Young Lee, MD, PhD^c,d^, Tae-Jin Youn, MD, PhD^b,c^,

In-Ho Chae, MD, PhD^b,c^, Cheol-Ho Kim, MD, PhD^b,c^

^a^Department of Cardiology, Wonju Medical College, Yonsei University, Wonju, Korea; ^b^Cardiovascular Center, Seoul National University Bundang Hospital, Seongnam-si, Korea; ^c^Department of Internal Medicine, Seoul National University, Seoul, Korea; ^d^Cardiovascular Center, Seoul National University Hospital, Seoul, Korea

1. **Supplementary Tables**

**Supplementary Table S1.** Brief comparison of the 2017 ACC/AHA hypertension guidelines and the JNC8 panel member report

|  | **2017 ACC/AHA guidelines** | **JNC8 panel member report** |
| --- | --- | --- |
| Definition of hypertension | SBP ≥130 mm Hg or DBP ≥80 mm Hg | SBP ≥140 mm Hg or DBP ≥90 mm Hg |
| Recommended pharmacologic treatment | - General population: SBP ≥140 mm Hg or DBP ≥90 mm Hg - DM, CKD: SBP ≥130 mm Hg or DBP ≥80 mm Hg - Presence of CVD or 10-year ASCVD risk ≥10%: SBP ≥130 mm Hg or DBP ≥80 mm Hg - Elderly (≥65 years of age without DM or CKD): SBP ≥130 mm Hg | - General population: SBP ≥140 mm Hg and DBP ≥90 mm Hg - Elderly (≥60 years of age without DM or CKD): SBP ≥150 mm Hg |
| Target BP goal | - General population: SBP <130 mm Hg and DBP <80 mm Hg - DM, CKD: SBP <130 mm Hg and DBP <80 mm Hg - Elderly (≥65 years of age without DM or CKD): SBP <130 mm Hg | - General population: SBP <140 mm Hg and DBP <90 mm Hg - Elderly (≥60 years of age without DM or CKD): SBP <150 mm Hg |

2017 ACC/AHA indicates the 2017 American College of Cardiology/American Heart Association Guideline for the Prevention, Detection, Evaluation and Management of High Blood Pressure in Adults; JNC8, Evidence-Based Guideline for the Management of High Blood Pressure in Adults: Report from the Panel Members Appointed to the Eighth Joint National Committee; SBP, systolic blood pressure; DBP, diastolic blood pressure; CVD, cardiovascular disease; ASCVD, atherosclerotic cardiovascular disease; DM, diabetes mellitus; CKD chronic kidney disease.

**Supplementary Table S2.** The prevalence of hypertension and the percentage of the population requiring pharmacologic antihypertensive treatment according to the 2017 ACC/AHA guideline and the JNC8 panel member report

|  | Prevalence of Hypertension | | |  | Requiring Antihypertensive Treatment | | |
| --- | --- | --- | --- | --- | --- | --- | --- |
|  | 2017 ACC/AHA | JNC8 | Differences |  | 2017 ACC/AHA | JNC8 | Differences |
| Overall (%) | 49.2 ± 0.6 | 30.4 ± 0.6 | 18.8 ± 0.44 |  | 35.3 ± 0.6 | 29.4 ± 0.5 | 6.0 ± 0.2 |
| Estimated number (million) | 14.7 | 9.1 | 5.6 |  | 10.6 | 8.8 | 1.8 |
| Sex (%) |  |  |  |  |  |  |  |
| Men | 57.7 ± 0.8 | 33.7 ± 0.8 | 23.9 ± 0.7 |  | 41.1 ± 0.8 | 32.9 ± 0.8 | 8.3 ± 0.4 |
| Women | 41.5 ± 0.7 | 27.4 ± 0.6 | 14.0 ± 0.4 |  | 30.2 ± 0.7 | 26.2 ± 0.6 | 4.0 ± 0.2 |
| Age group (%) |  |  |  |  |  |  |  |
| 30‒39 years | 27.7 ± 1.1 | 8.6 ± 0.6 | 19.1 ± 1.0 |  | 9.6 ± 0.7 | 8.6 ± 0.6 | 1.0 ± 0.2 |
| 40‒49 years | 41.9 ± 1.0 | 19.4 ± 0.9 | 22.6 ± 0.9 |  | 22.9 ± 0.9 | 19.4 ± 0.9 | 3.5 ± 0.4 |
| 50‒59 years | 55.9 ± 1.1 | 33.9 ± 1.0 | 22.0 ± 0.9 |  | 39.9 ± 1.1 | 33.9 ± 1.0 | 5.9 ± 0.5 |
| 60‒69 years | 64.4 ± 1.1 | 49.7 ± 1.2 | 14.6 ± 0.8 |  | 59.0 ± 1.2 | 46.3 ± 1.2 | 12.7 ± 0.8 |
| 70+ years | 73.7 ± 1.1 | 64.5 ± 1.2 | 9.2 ± 0.7 |  | 73.3 ± 1.1 | 60.2 ± 1.3 | 13.1 ± 0.8 |
| Area of residence (%) |  |  |  |  |  |  |  |
| Urban area | 48.2 ± 0.7 | 29.5 ± 0.6 | 18.7 ± 0.5 |  | 34.3 ± 0.7 | 28.6 ± 0.6 | 5.7 ± 0.2 |
| Rural area | 53.7 ± 1.5 | 34.5 ± 1.4 | 19.2 ± 1.0 |  | 40.6 ± 1.6 | 33.1 ± 1.4 | 7.5 ± 0.7 |
| Income quartiles (%) |  |  |  |  |  |  |  |
| Highest | 50.7 ± 1.1 | 32.4 ± 1.0 | 18.3 ± 0.8 |  | 37.6 ± 1.0 | 31.2 ± 1.0 | 6.4 ± 0.5 |
| Upper middle | 50.1 ± 1.1 | 31.3 ± 1.0 | 18.8 ± 0.8 |  | 36.6 ± 1.1 | 30.4 ± 1.0 | 6.2 ± 0.5 |
| Lower middle | 47.8 ± 1.0 | 29.5 ± 0.9 | 18.3 ± 0.8 |  | 34.0 ± 1.0 | 28.4 ± 0.9 | 5.6 ± 0.4 |
| Lowest | 48.0 ± 1.1 | 28.7 ± 1.0 | 19.4 ± 0.8 |  | 33.6 ± 1.1 | 27.8 ± 1.0 | 5.8 ± 0.5 |
| Education levels (%) |  |  |  |  |  |  |  |
| Primary school | 69.0 ± 1.0 | 55.0 ± 1.1 | 14.0 ± 0.7 |  | 63.3 ± 1.1 | 51.8 ± 1.1 | 11.5 ± 0.6 |
| Middle school | 57.1 ± 1.4 | 38.3 ± 1.4 | 18.8 ± 1.2 |  | 45.3 ± 1.5 | 36.9 ± 1.4 | 8.4 ± 0.9 |
| High school | 45.7 ± 1.0 | 25.4 ± 0.8 | 20.3 ± 0.8 |  | 29.5 ± 0.9 | 24.9 ± 0.8 | 4.6 ± 0.4 |
| University/college | 38.0 ± 1.0 | 17.6 ± 0.7 | 20.3 ± 0.8 |  | 21.0 ± 0.8 | 17.5 ± 0.7 | 3.5 ± 0.3 |

Values are presented as mean ± standard error. 2017 ACC/AHA indicates the 2017 American College of Cardiology/American Heart Association Guideline for the Prevention, Detection, Evaluation and Management of High Blood Pressure in Adults; JNC8 indicates the Evidence-Based Guideline for the Management of High Blood Pressure in Adults: Report from the Panel Members Appointed to the Eighth Joint National Committee.

**Supplementary Table S3.** Baseline characteristics of the study population according to blood pressure category and use of antihypertensive medications

|  | SBP/DBP (mm Hg) categories with no antihypertensive medication | | | | Taking antihypertensive medication |
| --- | --- | --- | --- | --- | --- |
|  | <120/80 | 120-129/<80 | 130-139/80-89 | ≥140/90 |  |
| Proportion (%) | 45.2% | 5.5% | 18.8% | 10.8% | 19.6% |
| Estimated number (million) | 13.5 | 1.7 | 5.6 | 3.2 | 5.9 |
| Age, years | 47.9 ± 0.2 | 48.6 ± 0.2 | 50.3 ± 0.3 | 52.5 ± 0.4 | 63.8 ± 0.3 |
| Sex, % |  |  |  |  |  |
| Men | 43.3 ± 0.5 | 46.2 ± 0.5 | 62.6 ± 0.8 | 65.3 ± 1.4 | 46.5 ± 1.0 |
| Women | 56.7 ± 0.5 | 53.8 ± 0.5 | 37.4 ± 0.8 | 34.7 ± 1.4 | 53.5 ± 1.0 |
| Anthropometric measurements |  |  |  |  |  |
| Body mass index, kg/m^2^ | 23.2 ± 0.0 | 23.6 ± 0.0 | 24.7 ± 0.1 | 25.1 ± 0.1 | 25.3 ± 0.1 |
| Waist circumference, cm | 79.9 ± 0.1 | 80.9 ± 0.1 | 84.3 ± 0.2 | 85.6 ± 0.3 | 86.8 ± 0.2 |
| SBP, mmHg | 110.0 ± 0.2 | 115.9 ± 0.2 | 130.0 ± 0.3 | 142.2 ± 0.4 | 128.6 ± 0.4 |
| DBP, mmHg | 71.5 ± 0.1 | 74.9 ± 0.2 | 85.6 ± 0.2 | 91.0 ± 0.3 | 76.3 ± 0.3 |
| Heart rate, beat / min | 56.8 ± 0.5 | 57.0 ± 0.5 | 57.5 ± 1.0 | 57.7 ± 1.6 | 58.4 ± 0.8 |
| Lifestyle behaviors |  |  |  |  |  |
| Regular physical activity, % | 33.8 ± 1.2 | 34.2 ± 1.1 | 36.7 ± 1.5 | 39.2 ± 2.7 | 34.4 ± 1.6 |
| Heavy alcohol drinking, % | 13.1 ± 0.5 | 16.5 ± 0.6 | 24.4 ± 1.0 | 30.7 ± 1.7 | 18.2 ± 1.2 |
| Daily calorie intake, kcal | 2052.4 ± 14.0 | 2083.0 ± 13.1 | 2195.4 ± 22.8 | 2191.2 ± 36.2 | 1822.6 ± 19.2 |
| Daily sodium consumption, mg | 4051.5 ± 44.3 | 4081.3 ± 42.3 | 4334.7 ± 78.9 | 4267.4 ± 90.8 | 3472.4 ± 58.4 |
| Comorbidities, % |  |  |  |  |  |
| Obesity | 25.4 ± 0.6 | 29.6 ± 0.5 | 42.7 ± 0.9 | 48.9 ± 1.5 | 50.7 ± 1.0 |
| Diabetes mellitus | 6.9 ± 0.3 | 7.4 ± 0.3 | 9.8 ± 0.6 | 10.2 ± 1.0 | 27.5 ± 1.1 |
| Dyslipidemia | 30.2 ± 0.6 | 32.8 ± 0.6 | 42.5 ± 1.0 | 45.0 ± 1.6 | 58.6 ± 1.1 |
| Laboratory findings |  |  |  |  |  |
| Total cholesterol, mg/dL | 190.3 ± 0.5 | 192.5 ± 0.5 | 199.7 ± 0.7 | 201.4 ± 1.3 | 185.3 ± 0.8 |
| HDL cholesterol, mg/dL | 51.4 ± 0.2 | 51.3 ± 0.1 | 49.7 ± 0.2 | 49.5 ± 0.4 | 47.8 ± 0.3 |
| LDL cholesterol, mg/dL | 117.1 ± 0.7 | 117.7 ± 0.6 | 121.5 ± 1.0 | 120.1 ± 1.5 | 107.7 ± 1.0 |
| Triglyceride, mg/dL | 130.7 ± 1.5 | 139.9 ± 1.5 | 171.1 ± 2.9 | 187.8 ± 5.2 | 159.3 ± 2.8 |
| Fasting glucose, mg/dL | 97.8 ± 0.3 | 98.8 ± 0.3 | 102.8 ± 0.5 | 104.2 ± 0.8 | 109.7 ± 0.6 |
| Calculated GFR (mL/min/1.73m^2^) | 94.9 ± 0.3 | 94.7 ± 0.3 | 93.0 ± 0.3 | 92.2 ± 0.6 | 84.7 ± 0.4 |
| 10-year ASCVD risk | 4.9 ± 0.1 | 5.8 ± 0.1 | 8.2 ± 0.2 | 11.0 ± 0.4 | 19.6 ± 0.4 |
| History of CVD | 2.4 ± 0.2 | 2.3 ± 0.2 | 2.4 ± 0.2 | 2.4 ± 0.4 | 12.9 ± 0.7 |

Values are presented as mean ± SE. ASCVD: atherosclerotic cardiovascular disease; CVD: cardiovascular disease; DBP: diastolic blood pressure; GFR: glomerular filtration rate; HDL: high density lipoprotein; LDL: low density lipoprotein; SBP: systolic blood pressure.

**Supplementary Table S4.** Hypertension control among all hypertensive subjects according to different target goals

|  | 2017 ACC/AHA | JNC8 |
| --- | --- | --- |
| Overall (%) | 16.1±0.5 | 59.5±1.0 |
| Sex (%) |  |  |
| Men | 12.8±0.7 | 51.3±1.4 |
| Women | 20.3±0.8 | 68.8±1.2 |
| Age group (%) |  |  |
| 30‒39 years | 1.0±0.3 | 14.8±2.8 |
| 40‒49 years | 5.5±0.8 | 34.8±2.5 |
| 50‒59 years | 12.3±0.9 | 51.4±1.9 |
| 60‒69 years | 27.8±1.2 | 74.9±1.4 |
| 70+ years | 32.5±1.3 | 79.9±1.1 |
| Area of residence (%) |  |  |
| Urban area | 15.7±0.6 | 58.7±1.1 |
| Rural area | 17.6±1.2 | 62.5±2.4 |
| Income quartiles (%) |  |  |
| Highest | 17.7±1.0 | 60.0±1.7 |
| Upper middle | 14.2±0.9 | 57.5±1.9 |
| Lower middle | 16.0±1.0 | 60.5±1.9 |
| Lowest | 16.4±1.1 | 60.0±2.0 |
| Education levels (%) |  |  |
| Primary school | 26.2±1.0 | 72.7±1.3 |
| Middle school | 19.0±1.3 | 62.2±2.2 |
| High school | 13.0±0.9 | 55.7±1.9 |
| University/college | 7.1±0.7 | 38.3±2.2 |

Values were presented as mean ± standard error. 2017 ACC/AHA indicates the 2017 American College of Cardiology/American Heart Association Guideline for the Prevention, Detection, Evaluation and Management of High Blood Pressure in Adults; JNC8, Evidence-Based Guideline for the Management of High Blood Pressure in Adults: Report from the Panel Members Appointed to the Eighth Joint National Committee.

**Supplementary Table S5.** Sensitivity analysis: baseline risk factors of study subjects who are taking antihypertensive medication stratified by the level of blood pressure (BP) control

|  | All adults with hypertension | Below 2017 ACC/AHA target | Below JNC8 but above 2017 ACC/AHA target | Above JNC8 target | P value |
| --- | --- | --- | --- | --- | --- |
| Number of subjects | 231,698 (100%) | 47,954 (20.7%) | 82,717 (35.7%) | 101,027 (43.6%) |  |
| Age, years | 58.1 ± 9.5 (231,698) | 58.5 ± 9.4 (47,954) | 58.8 ± 9.6 (82,717) | 57.4 ± 9.5 (101,027) | <0.001 |
| Female sex, % | 43.2% (100,060/231,698) | 45.7% (21,915/47,954) | 43.8% (36,271/82,717) | 41.4% (41,874/101,027) | <0.001 |
| Body mass index, kg/m^2^ | 24.6 ± 3.0 (231,594) | 23.9 ± 2.9 (47,931) | 24.5 ± 2.9 (82,690) | 25.0 ± 3.0 (100,973) | <0.001 |
| Blood pressure |  |  |  |  |  |
| Baseline SBP, mmHg | 143.3 ± 13.7 (204,549) | 139.1 ± 11 (30,632) | 139.1 ± 11.1 (73,989) | 147.8 ± 14.7 (99,928) | <0.001 |
| Baseline DBP, mmHg | 89.3 ± 8.8 (204,549) | 86.7 ± 7.3 (30,632) | 86.4 ± 7.4 (73,989) | 92.1 ± 9.1 (99,928) | <0.001 |
| Achieved SBP, mmHg | 134.7 ± 18.1 (231,698) | 113.7 ± 8.8 (47,954) | 129.1 ± 8.1 (82,717) | 149.1 ± 14.7 (101,027) | <0.001 |
| Achieved DBP, mmHg | 83.3 ± 11.6 (231,698) | 69.3 ± 5.7 (47,954) | 80.1 ± 4.7 (82,717) | 92.7 ± 9.2 (101,027) | <0.001 |
| Comorbidities, % |  |  |  |  |  |
| Diabetes | 8.5% (19,605/231,698) | 9.7% (4,638/47,954) | 7.5% (6,230/82,717) | 8.6% (8,737/101,027) | <0.001 |
| Dyslipidemia | 19.3% (44,689/231,698) | 25.9% (12,431/47,954) | 20.1% (16,641/82,717) | 15.5% (15,617/101,027) | <0.001 |
| Current smoking | 19.6% (43,452/221,761) | 18.7% (8,593/46,011) | 18.7% (14,833/79,169) | 20.7% (20,026/96,581) | <0.001 |
| Malignancy | 4.3% (10,025/231,698) | 6.9% (3,300/47,954) | 4.7% (3,876/82,717) | 2.8% (2,849/101,027) | <0.001 |
| Renal disease | 0.6% (1,498/231,698) | 0.9% (447/47,954) | 0.6% (493/82,717) | 0.6% (558/101,027) | <0.001 |
| Liver disease | 0.4% (915/231,698) | 0.7% (315/47,954) | 0.4% (342/82,717) | 0.3% (258/101,027) | <0.001 |
| Chronic pulmonary disease | 25.8% (59,668/231,698) | 34.6% (16,597/47,954) | 27.6% (22,846/82,717) | 20.0% (20,225/101,027) | <0.001 |
| Rheumatic disease | 6.5% (15,050/231,698) | 9.0% (4,337/47,954) | 6.9% (5,720/82,717) | 4.9% (4,993/101,027) | <0.001 |
| Laboratory findings |  |  |  |  |  |
| Total cholesterol, mg/dL | 202.2 ± 38.7 (231,343) | 196.4 ± 37.7 (47,867) | 201.5 ± 38.3 (82,601) | 205.5 ± 39.3 (100,875) | <0.001 |
| HDL cholesterol, mg/dL | 115.2 ± 38.2 (204,692) | 115.6 ± 37.4 (43,448) | 115.9 ± 37.8 (74,055) | 114.4 ± 39.0 (87,189) | <0.001 |
| LDL cholesterol, mg/dL | 148 ± 94.2 (205,810) | 137.0 ± 86.3 (43,622) | 146.2 ± 91.9 (74,439) | 155.1 ± 99.1 (87,749) | <0.001 |
| Triglyceride, mg/dL | 53.8 ± 28.7 (205,978) | 54.1 ± 28.4 (43,645) | 53.9 ± 28.7 (74,499) | 53.7 ± 28.9 (87,834) | 0.009 |
| Serum creatinine, mg/dL | 1.1 ± 1.3 (205,989) | 1.0 ± 1.1 (43,649) | 1.1 ± 1.2 (74,500) | 1.1 ± 1.4 (87,840) | <0.001 |

Values were presented as mean ± SD or percent. 2017 ACC/AHA indicates the 2017 American College of Cardiology/American Heart Association Guideline for the Prevention, Detection, Evaluation and Management of High Blood Pressure in Adults; JNC8, Evidence-Based Guideline for the Management of High Blood Pressure in Adults: Report from the Panel Members Appointed to the Eighth Joint National Committee; SBP, systolic BP; DBP, diastolic BP; HDL, high density lipoprotein; LDL, low density lipoprotein.

**Supplementary Table S6.** Sensitivity analysis: risk of cardiovascular events according to the level of blood pressure (BP) control among hypertensive subjects who were on antihypertensive medication

|  | **10-year event rates (%)** | | |  | **Unadjusted analysis** | |  | **Multivariable adjustment** | |  | **Propensity score matching analysis** | |
| --- | --- | --- | --- | --- | --- | --- | --- | --- | --- | --- | --- | --- |
|  | **Below 2017 ACC/AHA target** | **Below JNC8 but above 2017 ACC/AHA target** | **Above JNC 8 target** |  | **HR (95% CI)** | **p-value** |  | **HR (95% CI)** | **p-value** |  | **HR (95% CI)** | **p-value** |
| **2017 ACC/AHA goal vs. JNC8 goal** | |  |  |  |  |  |  |  |  |  |  |  |
| Composite cardiovascular evets | 8.60 | 10.27 |  |  | 0.84 (0.81-0.87) | <0.001 |  | 0.88 (0.84-0.92) | <0.001 |  | 0.92 (0.87-0.97) | 0.003 |
| Death from any cause | 5.93 | 6.53 |  |  | 0.91 (0.87-0.95) | <0.001 |  | 0.76 (0.71-0.80) | <0.001 |  | 0.78 (0.73-0.84) | <0.001 |
| Cardiac death | 1.33 | 1.58 |  |  | 0.84 (0.77-0.92) | <0.001 |  | 0.73 (0.64-0.83) | <0.001 |  | 0.81 (0.70-0.94) | 0.007 |
| Myocardial infarction | 1.88 | 2.15 |  |  | 0.87 (0.81-0.94) | <0.001 |  | 0.95 (0.86-1.04) | 0.281 |  | 0.95 (0.85-1.06) | 0.330 |
| Stroke | 3.98 | 5.22 |  |  | 0.76 (0.72-0.80) | <0.001 |  | 0.82 (0.77-0.87) | <0.001 |  | 0.88 (0.82-0.95) | <0.001 |
| Heart failure | 3.50 | 3.82 |  |  | 0.92 (0.86-0.97) | 0.003 |  | 0.93 (0.87-1.00) | 0.064 |  | 0.98 (0.90-1.07) | 0.598 |
|  |  |  |  |  |  |  |  |  |  |  |  |  |
| **2017 ACC/AHA goal vs. poorly controlled** | |  |  |  |  |  |  |  |  |  |  |  |
| Composite cardiovascular evets | 8.60 |  | 12.28 |  | 0.70 (0.67-0.72) | <0.001 |  | 0.72 (0.69-0.75) | <0.001 |  | 0.74 (0.70-0.77) | <0.001 |
| Death from any cause | 5.93 |  | 7.61 |  | 0.78 (0.75-0.81) | <0.001 |  | 0.57 (0.54-0.61) | <0.001 |  | 0.61 (0.57-0.65) | <0.001 |
| Cardiac death | 1.33 |  | 2.01 |  | 0.66 (0.60-0.72) | <0.001 |  | 0.53 (0.47-0.61) | <0.001 |  | 0.57 (0.49-0.65) | <0.001 |
| Myocardial infarction | 1.88 |  | 2.49 |  | 0.76 (0.70-0.82) | <0.001 |  | 0.82 (0.74-0.89) | <0.001 |  | 0.84 (0.75-0.93) | 0.001 |
| Stroke | 3.98 |  | 6.63 |  | 0.60 (0.57-0.63) | <0.001 |  | 0.67 (0.63-0.71) | <0.001 |  | 0.67 (0.63-0.72) | <0.001 |
| Heart failure | 3.50 |  | 4.26 |  | 0.82 (0.78-0.87) | <0.001 |  | 0.77 (0.72-0.83) | <0.001 |  | 0.78 (0.72-0.85) | <0.001 |

2017 ACC/AHA indicates the 2017 American College of Cardiology/American Heart Association Guideline for the Prevention, Detection, Evaluation and Management of High Blood Pressure in Adults; JNC8, Evidence-Based Guideline for the Management of High Blood Pressure in Adults: Report from the Panel Members Appointed to the Eighth Joint National Committee.

1. **Supplementary Figures**

**Supplementary Figure S1.** Indications accounting for the increase in the number of subjects who are recommended antihypertensive pharmacologic treatment by the 2017 ACC/AHA guidelines but not by the JNC8 report

**
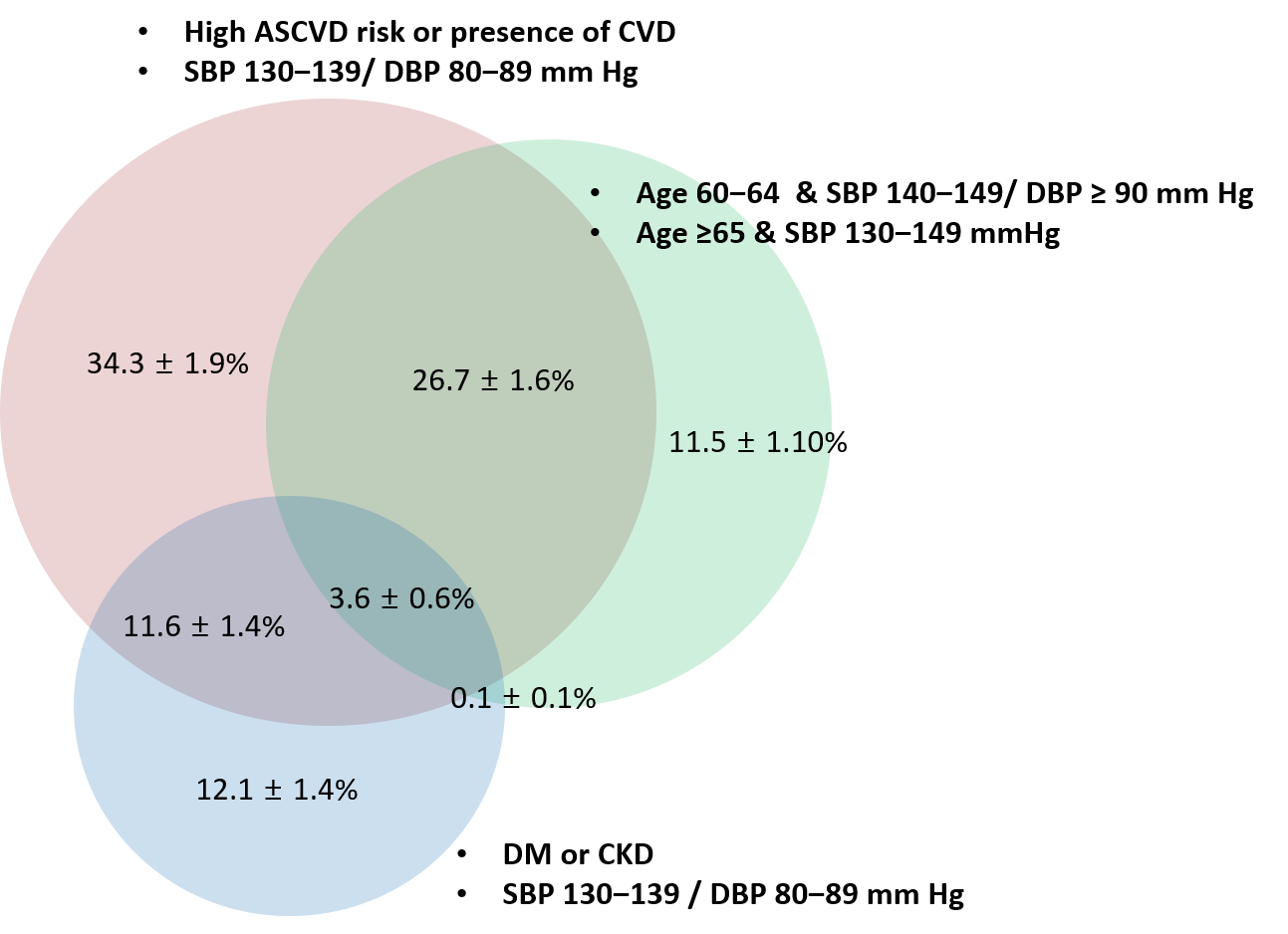
**

**Supplementary Figure S2.** Kaplan-Meier survival curve free from major cardiovascular events among hypertensive subjects according to their achieved blood pressure: (left) systolic blood pressure, and (right) diastolic blood pressure

| 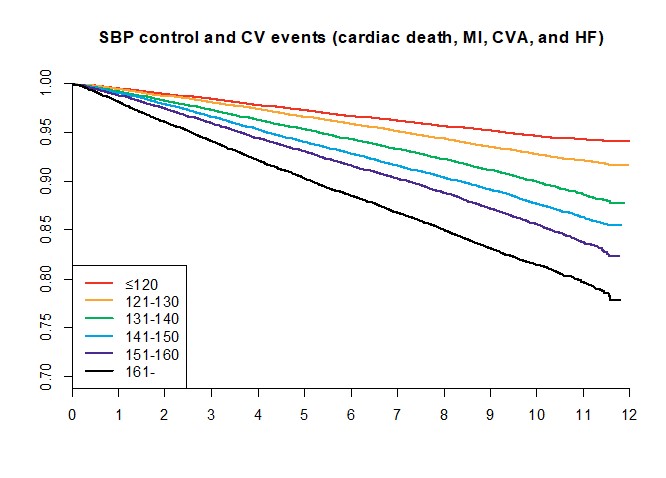 | 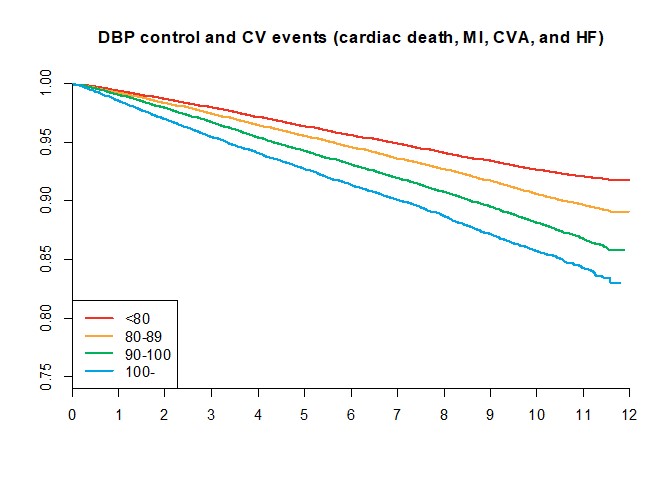 |
| --- | --- |

**Supplementary Figure S3.** Comparison of differences in baseline variables before and after propensity score matching

**
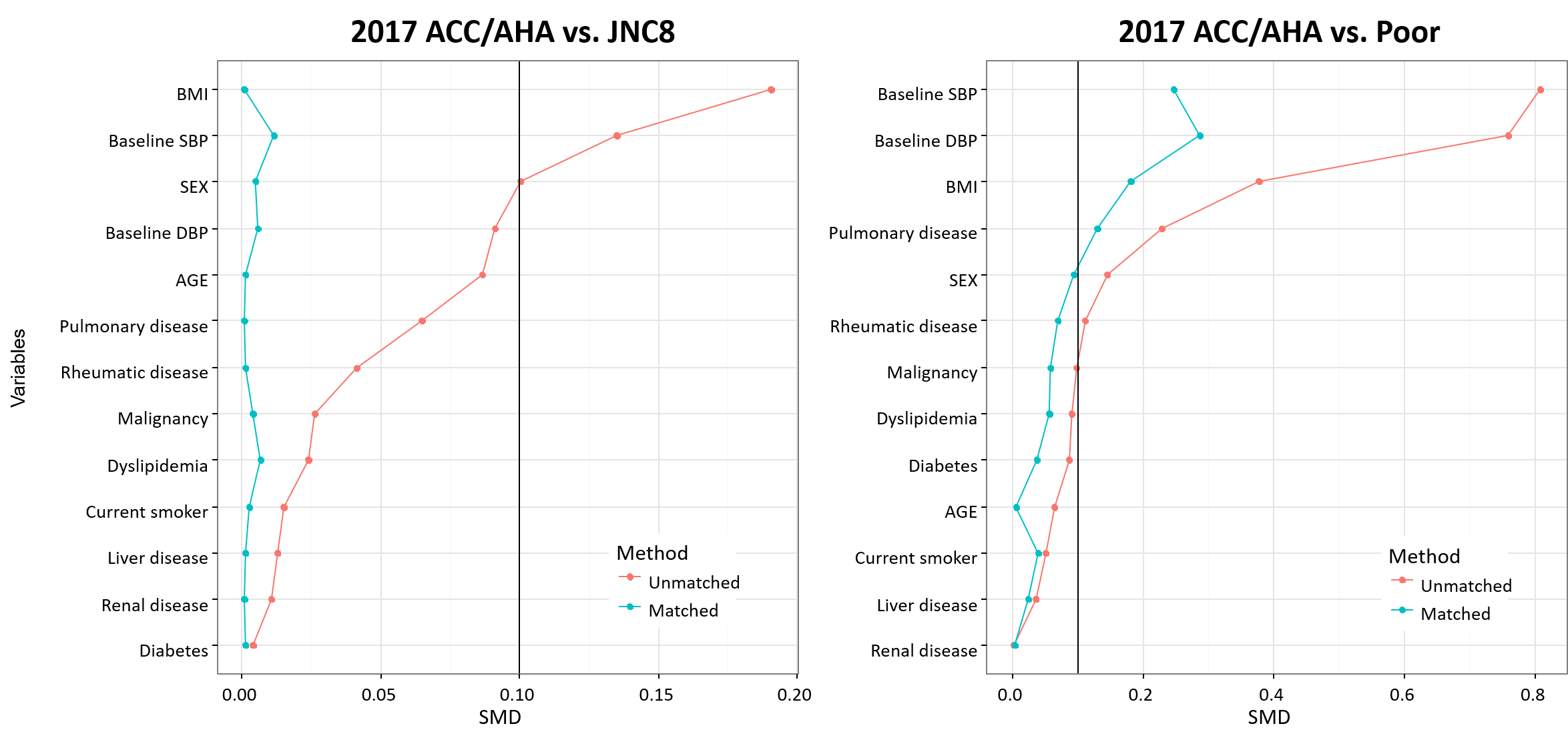
**

**Supplementary Figure S4.** Subgroup analysis for the risk of major cardiovascular events according to the achievement of blood pressure target goals


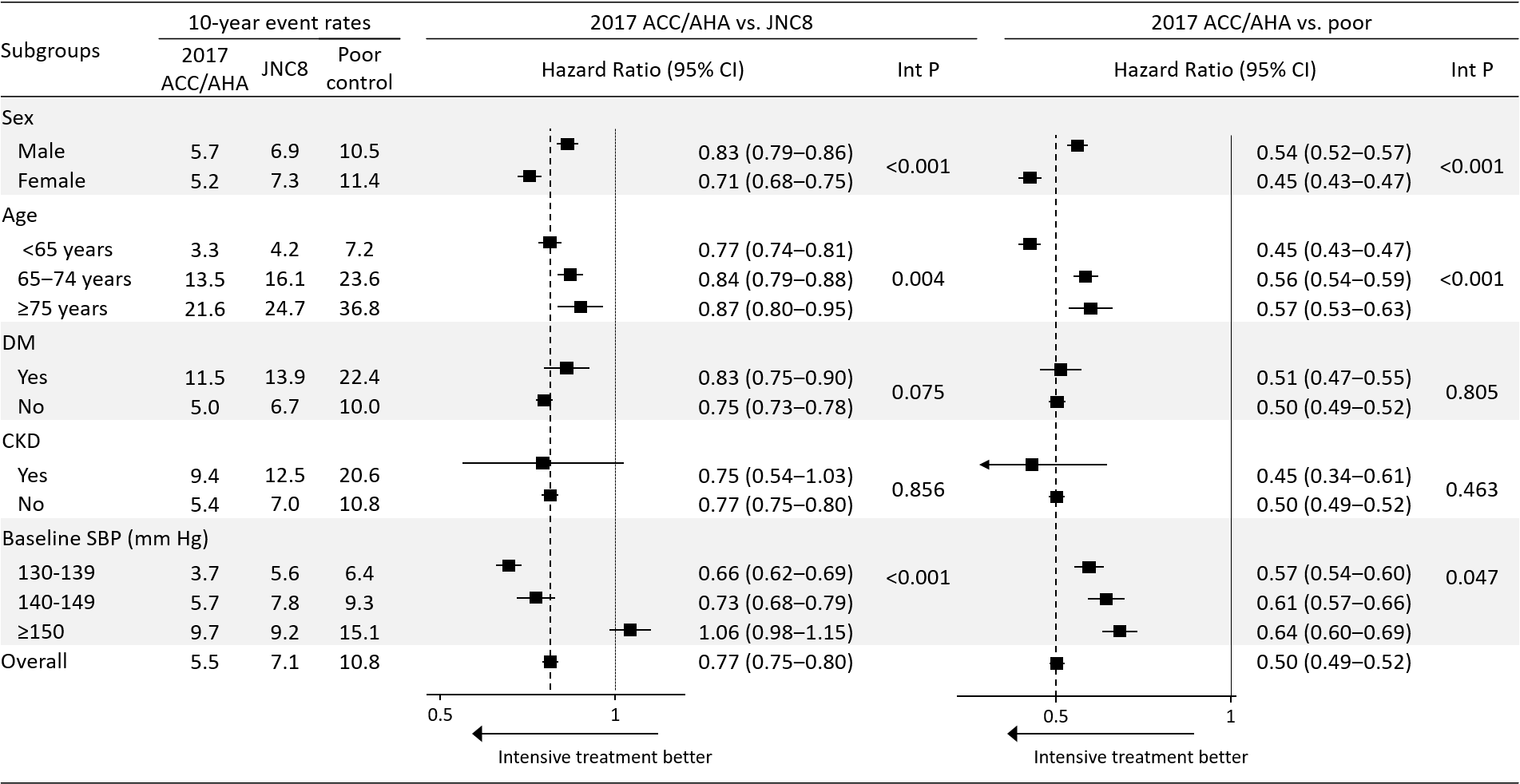


**Supplementary Figure S5.** Sensitivity analysis: Kaplan-Meier survival curve free from major cardiovascular events among hypertensive subjects who were on antihypertensive medication according to their achieved blood pressure


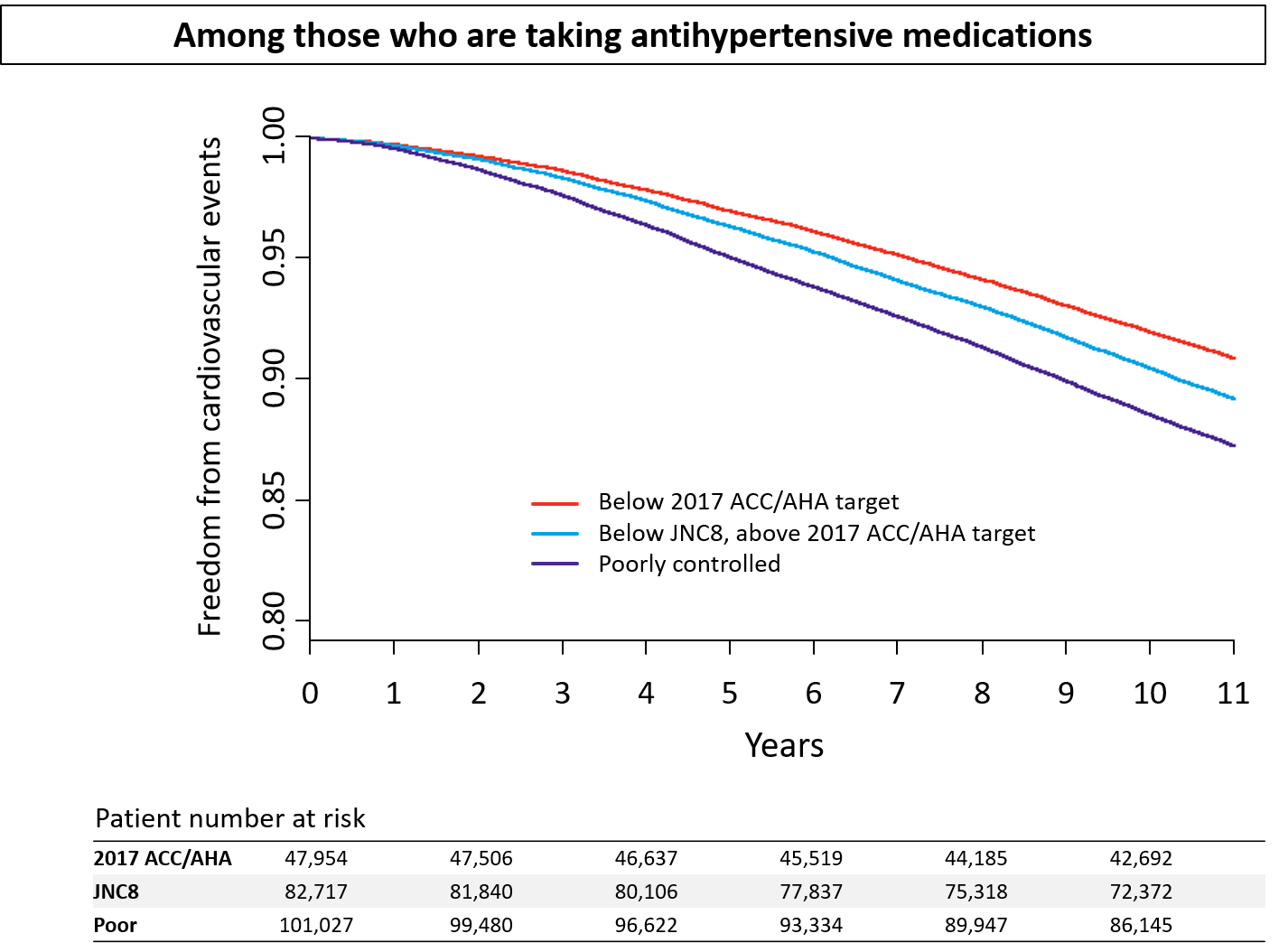

Supplement: Supplementary file 1 — Supplementary information [file 41598_2018_31549_MOESM1_ESM.docx]
